# The Effect of “Offline-to-Online” Trust Transfer on the Utilization of Online Medical Consultation Among Chinese Rural Residents: Experimental Study

**DOI:** 10.2196/43430

**Published:** 2023-12-26

**Authors:** Jiao Lu, Jingyan Bai, Heng Zhao, Xiaoxiao Zhang

**Affiliations:** 1 School of Public Policy and Administration Xi’an Jiaotong University Xi’an, Shaanxi China; 2 School of Public Health Shanxi Medical University Taiyuan, Shanxi China; 3 Academy of Medical Sciences Shanxi Medical University Taiyuan, Shanxi China

**Keywords:** "offline-to-online" trust transfer, online medical consultation, health care services utilization, Chinese rural residents, direct and family spillover effects

## Abstract

**Background:**

Online medical consultation can serve as a valuable means for rural residents to access high-quality health care resources, thereby mitigating the geographic and economic disadvantages prevalent in rural areas. Nevertheless, due to lower cognitive abilities, rural residents often face challenges in trusting and making effective use of online medical consultations. More likely, adopting a bounded rational decision-making model that facilitates the “offline-to-online” trust transfer could prove to be a potentially effective approach. This strategy aims to encourage less technologically experienced rural residents to trust and make use of online medical consultations.

**Objective:**

This study aims to characterize the status of “offline-to-online” trust transfer among rural residents in the context of internet health care, and analyze its direct impact on facilitating the utilization of online medical consultation. Additionally, we investigate the family spillover effect of “offline-to-online” trust transfer in promoting the use of online medical consultation among rural family members, considering its distributional effect across various education levels of the population.

**Methods:**

A multistage stratified random sampling method was used to survey participants in rural areas of China from July to September 2021, encompassing a total of 2597 rural residents from 960 rural households. Propensity score values were estimated using logit regression, and the propensity score matching method, using the K-nearest neighbor matching, radius matching, and kernel matching methods, was applied to create matched treatment and control samples of rural residents based on their experience of “offline-to-online” trust transfer. Subsequently, we calculated average treatment effect scores to compare the differences in utilizing online medical consultation between the treatment and control rural samples.

**Results:**

As many as 551/960 (57.4%) rural residents experienced an “offline-to-online” trust transfer, with a higher likelihood observed in the older population with lower levels of education and higher satisfaction with local health care services. Furthermore, rural residents who underwent “offline-to-online” trust transfer were 37%-40% more likely to utilize online medical consultation compared with those who did not experience this trust transfer. Additionally, family members of householders who underwent “offline-to-online” trust transfer were 25%-28% more likely to utilize online medical consultation than those whose householders did not experience this trust transfer. Notably, when compared with populations with high-level education, the “offline-to-online” trust transfer had more significant direct and spillover effects on the utilization of online medical consultation services among rural residents with low-level education.

**Conclusions:**

To enhance the “offline-to-online” trust transfer among rural residents and its facilitation in their utilization of online medical consultation, as well as other mobile health (mHealth) and ubiquitous health (uHealth) services, we recommend that online health care providers adopt a “patient-oriented” service model. This approach aims to elevate rural residents’ satisfaction with local health care services and harness the trust-building functions inherent in physician-patient relationships and among family members.

## Introduction

### Background

The urban-rural system in China has led to challenges for rural residents in accessing and utilizing quality health care resources compared with their urban counterparts. This discrepancy arises from the relative disadvantages faced by rural residents in both geographical [1,2] and financial [3,4] aspects, resulting in a shortage of diagnostic and therapeutic equipment [5] and a lack of high-quality health care professionals [6]. Therefore, there is a need to make concerted efforts to enhance the accessibility of health care services in rural areas of China [7,8]. Coincidentally, online medical consultation, a crucial component of mobile health (mHealth) and ubiquitous health (uHealth) services, plays a pivotal role. Through this service, health care professionals provide online medical information, counseling, disease diagnosis, and treatment services to patients on third-party digital platforms [9,10]. This approach has demonstrated its effectiveness in improving access to quality health care services for rural residents [11], overcoming constraints related to time and space [12,13]. However, previous studies have revealed that, despite the rapid growth of online medical consultation during the COVID-19 pandemic, rural residents were nearly 2 times as likely to face insufficient access to online health care services compared with their urban counterparts (9.1% vs 5.4%) [14]. This discrepancy can likely be attributed to the fact that rural residents, primarily comprising middle-aged and older individuals, often exhibit a more traditional mindset and may face challenges in adapting to new technologies [15-17]. This reluctance to embrace technological advancements makes it challenging for them to place trust in online medical consultation [17,18], consequently hindering their ability to fully leverage this service for accessing quality health care [19,20]. Therefore, it is crucial to focus on building trust to encourage rural residents to utilize online medical consultations [21].

The concept of heuristic systems influencing individual decision-making suggests that individuals with lower cognitive abilities are more likely to establish trust through trust transfer [22]. For rural residents, especially middle-aged and older individuals with lower learning abilities [15], trust transfer may be a suitable approach for building trust in online medical consultation. Trust transfer refers to the phenomenon of placing trust in an unfamiliar object based on the trust one has for a known object that is somehow linked to the unfamiliar one [23]. In practical terms, owing to the trust rural residents have in offline physicians, they may establish trust in online medical consultation services by transferring their trust from offline physicians to the online medical consultation services recommended or provided by these physicians (ie, “offline-to-online” trust transfer). While some existing research suggests that “offline-to-online” trust transfer is challenging in medical situations [24], an opposing viewpoint has been validated, indicating that patients’ trust in offline physicians can indeed bolster their trust in online medical services [25]. It is worth noting that Meng et al [26] uniquely exemplified the “offline-to-online” trust transfer phenomenon among older patients, specifically using mHealth services as an example and assessing the impact of this trust transfer on patients’ utilization of mHealth services [26]. Indeed, there is a shortage of studies that substantiate the feasibility of establishing trust through “offline-to-online” trust transfer among rural residents and its direct impact on encouraging their utilization of mHealth and uHealth services, among other aspects.

Moreover, humans inherently exhibit social attributes and are susceptible to the prevailing ideas in their surroundings. In Chinese culture, the concept of family is highly esteemed. The opinions and beliefs of key family members frequently shape the perspectives of other family members, leading to a phenomenon referred to as the “family spillover effect” [27]. Existing studies have affirmed the existence of the family spillover effect, particularly in the evaluation of the value of health care interventions [28] and the cost-effectiveness of medical treatments [29]. Furthermore, individuals do not solely rely on heuristic systems when making decisions; they are also influenced by analytical systems. The level of education serves as a notable indicator of an individual’s analytical ability. Past research has demonstrated that individuals with higher levels of education are more likely to embrace emerging services, such as eHealth services [30]. In essence, the adoption of emerging services by individuals exhibits a distributional effect across populations with varying levels of education. Nevertheless, the current literature, while examining the impact of trust transfer on health care services utilization, overlooks the influence of individuals’ social attributes and the interaction between heuristic systems and analytical systems in their decision-making processes. This oversight leads to a failure to account for the family spillover effect of “offline-to-online” trust transfer and its distributional impact on populations with different levels of education. To bridge these gaps, further research is imperative, aiming to offer a comprehensive understanding of how trust transfer influences the utilization of health care services among rural residents.

Furthermore, correlations between explanatory variables and error terms in the regression equation may arise due to the interconnection of variables, model error settings, sample truncation, and measurement error issues [31]. Such issues may give rise to endogeneity problems in the overall regression, potentially resulting in estimation errors. The challenge stems from the fact that the impact of the error term can induce a change in the dependent variable. However, we can only observe alterations in the explanatory and dependent variables, not the direct effect of the error term [32]. Consequently, during parameter estimation, the estimator might mistakenly attribute the change in the explanatory variable to the associated error term, even when the change is caused by the error term rather than the explanatory variable. This misattribution can lead to systematic overestimation or underestimation of the coefficients of the explanatory variables. By contrast, propensity score matching does not rely on explicit model-setting assumptions. It avoids endogeneity issues by constructing a comparable “control group” versus the “treatment group” within a counterfactual framework to estimate the treatment effect [33]. Consequently, we used the propensity score matching method to address the endogeneity challenge and estimate the actual effect of “offline-to-online” trust transfer on the utilization of online medical consultation.

Therefore, considering the reality that rural residents typically exhibit low cognitive levels and face challenges in quickly accepting emerging concepts [34], it becomes crucial to acknowledge the pivotal role of trust in motivating individuals to embrace emerging services. This recognition is especially important in light of the intrinsic influence of both heuristic and analytical systems on individual decision-making. This study centered around key inquiries within the framework of “offline-to-online” trust transfer. First, it explored the existence of an “offline-to-online” trust transfer phenomenon among rural residents concerning online medical consultation, examining its direct impact on promoting their utilization of online medical consultation. Second, the study delved into whether a family spillover effect of “offline-to-online” trust transfer exists, specifically in fostering the utilization of online medical consultation among rural residents. Furthermore, the study aimed to investigate whether there is a distributional effect of “offline-to-online” trust transfer on rural residents’ utilization of online medical consultation, with a specific focus on potential variations attributed to differences in individuals’ education levels.

### Theoretical Analysis

Bounded rationality emphasizes that human behavior is “consciously rational, but this rationality is limited.” This implies that individuals tend to make judgments based on rules of thumb, hopes, and beliefs rather than engaging in perfectly rational calculations [35]. At this juncture, the inherent uncertainty and risk associated with emerging services elevate the law of trust to a paramount criterion for individual decision-making. The law of trust serves as a decision-making simplification mechanism grounded in the bounded rationality exercised by individuals when confronted with future uncertainties and risks [36]. This is because trust plays a crucial role in aiding individuals to navigate the perception of uncertainty and risk linked with emerging services when making decisions, ultimately contributing to an enhanced utilization of online medical consultation [37]. According to the dual-channel model, the human cognitive decision model comprises 2 systems: the heuristic systems and the analytical systems [38]. These 2 systems represent the pathways individuals utilize to establish trust. Given their low-level learning ability, rural residents appear to lean toward finding solutions through intuition and relevant information matching, rather than making decisions through reasoning and logic when faced with problems [39]. Trust transfer effectively leverages this tendency to establish trust. Fundamentally, when the target and the trusted entity share contextual relevance or belongingness, trust can be transferred between the trusted entity and an unknown entity based on the association between the target entity and the trusted source entity [40,41]. According to the trust transfer mechanism, when an individual undergoes trust transfer, they might extend their trust to online medical services recommended or provided by an offline physician whom they consult frequently and trust. This phenomenon is referred to as “offline-to-online” trust transfer (Multimedia Appendix 1; also see [42-46]). As a result, individuals may extend their trust to online medical consultation and utilize these services in accordance with the principles outlined by the law of trust. Thus, we proposed the first hypothesis:

- *Hypothesis 1*: Rural residents are more likely to experience the “offline-to-online” trust transfer, and the “offline-to-online” trust transfer may have a direct effect on promoting their utilization of online medical consultation.

In line with the Unified Theory of Acceptance and Use of Technology, social influence is identified as a crucial factor directly impacting an individual’s intention to adopt emerging services. This influence is considered alongside performance expectancy and effort expectancy [47]. Social influence is defined as the degree to which an individual believes that other significant individuals expect them to use an emerging service [48]. This implies that an individual’s behavioral intention is shaped by the attitudes of those around them toward the emerging service. Among the 3 mechanisms through which social influence impacts an individual’s behavioral intention, the internalization and identification mechanisms emphasize the autonomy of the individual and exert a more significant social influence than the conformity mechanism, which implies a tendency toward pressure [49]. Undoubtedly, individuals embark on the process of socialization right from birth [50], continually influenced by society. The family systems theory [51], psychodynamic theory [52], and sociological theory [53] collectively acknowledge that the family stands as one of the primary and earliest media of socialization. It serves as a crucial arena for personal life and emotional communication for individuals, representing the most significant subsystem for generating social influence through the internalization and identification mechanisms. These mechanisms inevitably exert a profound impact on the emotions and behaviors of family members. Additionally, traditional Chinese Confucianism places a strong emphasis on family culture, suggesting that the opinions of family members play a pivotal role in the decision-making of individuals [54]. Therefore, the recommendations or views of family members are likely to have a positive impact on the willingness of rural residents to adopt emerging services [55], a phenomenon referred to as the family spillover effect. Furthermore, according to the dual-channel model, it is evident that individuals do not make decisions solely through heuristic systems; they are also influenced by analytical systems [56]. An individual’s cognitive ability serves as the primary channel of analytical systems, aiding them in building trust [57]. Education, to some extent, mirrors an individual’s perception of emerging concepts [32]. Therefore, education can prompt individual reflection and interact with the “offline-to-online” trust transfer initiated by heuristic systems. This interaction may result in varied utilization of online medical consultation among rural residents based on their different levels of education, a phenomenon referred to as a distributional effect. Thus, we proposed the second hypothesis:

- *Hypothesis 2*: The “offline-to-online” trust transfer may have a family spillover effect on promoting rural residents’ utilization of online medical consultation. Moreover, the “offline-to-online” trust transfer may have a distributional effect on promoting rural residents’ utilization of online medical consultation due to differences in their education levels.

## Methods

### Data Sources

**Figure 1:**
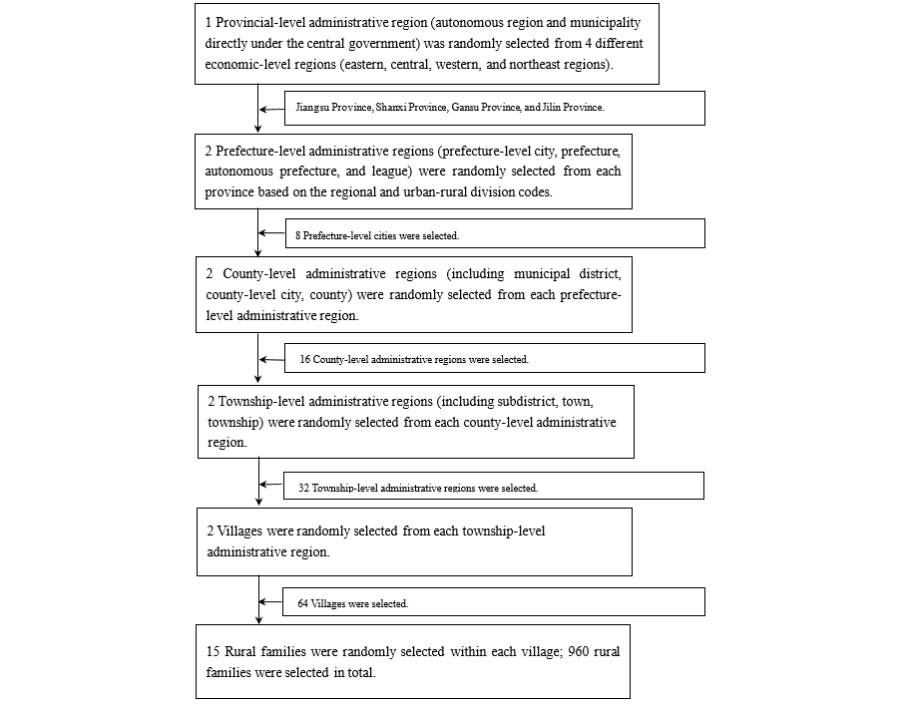
Flowchart of the sampling procedures. The Urban-Rural Division Codes comprise the standardized statistical division code, encompassing both urban and rural classifications. The “Statistical Division Code and Urban and Rural Division Codebase” has been established for the cohesive utilization across census, comprehensive statistics, sampling surveys, and special surveys. All sampling units within this codebase are assigned specific numbers. The League constitutes one of the historical administrative units of China, sharing administrative equivalence with prefecture-level cities, regions, and autonomous prefectures. Positioned within prefecture-level administrative regions, it stands as a distinctive administrative division within Inner Mongolia.

This study used a multistage stratified random sampling method using a random-number table to survey Chinese rural residents between July and September 2021. The sampling procedures are outlined in Figure 1.

### Ethics Approval and Considerations

Approval for the investigation was obtained from the Institutional Ethical Review Board of Xi’an Jiaotong University (approval number 2020-119), and informed consent was obtained from all participants. The survey encompassed a total of 960 rural families, with family members aged 18 years or older included in the data collection. A total of 2903 questionnaires were gathered, excluding those with missing key information or evidently incorrect details. Of these, 2597 were deemed valid, resulting in an effective rate of 89.46%.

### Variables

#### Independent Variable

The independent variable in this study is whether an individual has undergone an “offline-to-online” trust transfer. We utilized the trust transfer game to assess the status of an individual’s “offline-to-online” trust transfer, using a measurement method adapted from Delgado-Márquez et al [42]. In this study, each individual was tasked with participating in 3 simple decision-making tasks involving 3 types of health care service providers: the offline doctor with whom the individual is familiar (the trustee), the online medical platform where this physician worked or that the physician recommended (the third trustee), and the online medical platform where this physician neither worked nor recommended (the stranger).

The “offline-to-online” trust transfer is defined as the case where the truster is willing to pay money to the third trustee, and the amount is higher than the amount paid to the stranger. Furthermore, the trust transfer is maximized when the amount received by the third trustee is at least equal to the amount received by the trustee, signifying a complete “offline-to-online” trust transfer.

#### Dependent Variable

The assessment of individuals’ utilization of online medical consultation was based on the following question: “If necessary, would you use online medical consultation?” (yes=1, no=0).

#### Matching Variables

Drawing from previous online health literature [32,58], we identified 3 categories of matching variables (Table 1). The first category comprised sociodemographic characteristics of rural residents, encompassing variables such as sex, age, education, occupation, per capita disposable income, personal health status, reliance on the internet for health information, and whether they have acquaintances engaged in internet medical treatment. The second category involved rural residents’ home internet access, encompassing factors such as whether their home has Wi-Fi access and the number of computers and mobile phones in their home. The third category encompassed the health care environment of rural residents, incorporating variables such as the accessibility of health care services, satisfaction with offline medical treatment, and so forth.

**Table 1:** Variable definitions and descriptive statistics.

Variables				Values (N=2597)		
**Dependent variable**						
	**Online medical consultation, n (%)**					
		Use the online medical consultation=1	1405 (54.10)			
		Nonuse of the online medical consultation=0	1192 (45.90)			
**Independent variable**						
	“**Offline-to-online” trust transfer, n (%)**					
		Experienced trust transfer=1	1520 (58.53)			
		No experienced trust transfer=0	1077 (41.47)			
**Control variables**						
	**Sex, n (%)**					
		Man=1	1418 (54.60)			
		Woman=0	1179 (45.40)			
	Age (years), mean (SD)		44.807 (11.88)			
	Years of education (years), mean (SD)		8.947 (2.96)			
	**Occupation, n (%)**					
		Occupation is related to the internet=1	132 (5.08)			
		Occupation is not related to the internet=0	2465 (94.92)			
	Per capita disposable income by family year, mean (SD)		15,301.630 (6723.95)			
	**Personal health status, n (%)**					
		Poor=1	451 (17.37)			
		General=2	1131 (43.55)			
		Better=3	1015 (39.08)			
	**Home network access, n (%)**					
		With network connectivity=1	1647 (63.42)			
		Network not connected=0	950 (36.58)			
	Number of home mobile phones and computers (including tablets), mean (SD)		2.935 (0.984)			
	**Whether health information is obtained from the internet, n (%)**					
		Yes=1	1473 (56.72)			
		No=0	1124 (43.28)			
	**Whether recognize any acquaintances engaged in internet medicine, n (%)**					
		Recognize=1	36 (1.39)			
		Do not recognize=0	2561 (98.61)			
	Your distance to the nearest medical facility (hundred meters), mean (SD)		13.668 (7.011)			
	**Satisfaction with medical technology during a final offline visit with a physician, n (%)**					
		Total dissatisfaction=1	24 (0.92)			
		Less satisfied=2	491 (18.91)			
		General=3	899 (34.62)			
		More satisfied=4	947 (36.47)			
		Fully satisfied=5	236 (9.09)			
	**Satisfaction with the service attitude of the physician during the last offline visit, n (%)**					
		Total dissatisfaction=1	28 (1.08)			
		Less satisfied=2	167 (6.43)			
		General=3	818 (31.50)			
		More satisfied=4	1140 (43.90)			
		Fully satisfied=5	444 (17.10)			
	**Satisfaction with frequently visited medical institutions, n (%)**					
		Totally dissatisfied=1	8 (0.31)			
		Less satisfied=2	239 (9.20)			
		General=3	720 (27.72)			
		More satisfied=4	1363 (52.48)			
		Fully satisfied=5	267 (10.28)			

### Measurements

#### Sample Groups

The sample was bifurcated into a treated sample and a control sample based on whether individuals had undergone “offline-to-online” trust transfer. The treated sample comprised rural residents who had experienced “offline-to-online” trust transfer, while the control sample included rural residents who had not undergone “offline-to-online” trust transfer.

#### Adopted Methods

To mitigate selection bias among individuals, we used the propensity score matching method for random sample matching. Initially, we used logit regression to estimate propensity scores, selecting observable confounding variables for this purpose. In the second step, we matched the samples of rural residents in the treated and control groups using K-nearest neighbor matching, radius matching, and kernel matching. Subsequently, in the third step, we computed the average treatment effects on the treated (ATT) by comparing the utilization level of online medical consultation for the 2 samples based on the postmatch sample.

#### The Direct Effects of “Offline-to-Online” Trust Transfer on the Utilization of Online Medical Consultation of Rural Residents

We conducted a match analysis, utilizing whether an individual had experienced “offline-to-online” trust transfer as the matching categorical variable (yes=treated sample, no=control sample), to estimate the average treatment effect.

ATT_1_ = *E*(*Y*_1_*_i_* – *Y*_0i_|Demon*_i_*=1)

where Demon*_i_* = {0,1}. *Demon_i_* is a demonstrative pronoun that has no special meaning in and of itself. It indicates whether a rural resident has experienced “offline-to-online” trust transfer, where 0 is the control sample and 1 is the treatment sample; *Y*_1_ indicates whether a rural resident utilizes online medical consultation; *Y*_1_*_i_* denotes the utilization of online medical consultation of rural residents who experienced “offline-to-online” trust transfer and *Y*_0_*_i_* denotes the utilization of online medical consultation of rural residents who did not experience “offline-to-online” trust transfer. ATT_1_ represents the average processing effect of online medical consultation utilization of rural residents who experienced an “offline-to-online” trust transfer compared with those who did not experience an “offline-to-online” trust transfer.

#### The Family Spillover Effect of “Offline-to-Online” Trust Transfer on the Utilization of Online Medical Consultation of Rural Residents

In rural families, the “householder” plays an irreplaceable role in information acquisition, transmission, and health-related decision-making among family members [59]. Accordingly, we conducted an analysis of the family spillover effect of the “householder” on the utilization of online medical consultation by family members. This analysis was based on the assumption that “householders” experiencing significant “offline-to-online” trust transfer would have a direct impact on their utilization of online medical consultation. In accordance with Rosenthal and Marshall’s inquiry [60], we posed the question “Who is in charge of your family affairs?” to ascertain whether the individual could be identified as the “householder.”

The average treatment effect was estimated through a matching analysis, considering family members who experienced “offline-to-online” trust transfer as the treated sample and those who did not experience such trust transfer as the control sample.

ATT_2_ = *E*(*Y*_1_*_i_* – *Y*_0i_|Surr*_i_*=1)

where Surr*_i_*=1 represents the family member of the householder who experienced “offline-to-online” trust transfer, and Surr*_i_*=0 represents the householder who did not experience an “offline-to-online” trust transfer; *Y_i_* indicates the status of rural residents’ utilization of online medical consultation; *Y*_1_*_i_* denotes that the family members of the householder who experienced “online-to-offline” trust transfer have utilized the online medical consultation; and *Y*_0_*_i_* denotes that family members of the householder who did not experience an “online-to-offline” trust transfer have not utilized the online medical consultation; ATT_2_ denotes the average treatment effect of the “offline-to-online” trust transfer of family members whose householders experienced “offline-to-online” trust transfer on their utilization of online medical consultation compared with householders who did not experience “offline-to-online” trust transfer.

#### The Distributional Effect of “Offline-to-Online” Trust Transfer on the Utilization of Online Medical Consultation of Rural Residents

We categorized rural residents by educational level and examined the distributional effect of “offline-to-online” trust transfer on their utilization of online medical consultation among residents with varying education levels.

## Results

### Analysis of Sample Characteristics

As presented in Table 2, 551/960 (57.4%) rural householders experienced “offline-to-online” trust transfer and 926/969 (95.6%) of the family members of these householders also experienced this trust transfer. By contrast, among the 409 rural householders who did not experience “offline-to-online” trust transfer, approximately 36.2% (148/409) of their family members still experienced “offline-to-online” trust transfer. The utilization of online medical consultation among rural householders who experienced “offline-to-online” trust transfer was 4.93 times higher than that of rural householders who did not undergo such trust transfer. Additionally, the utilization of online medical consultation among family members who experienced “offline-to-online” trust transfer was 2.06 times higher compared with rural family members who did not experience “offline-to-online” trust transfer. Apart from the variable concerning whether a respondent recognizes the person engaged in internet-based medical care, there were significant differences in other observable covariates. These differences were observed between rural householders who experienced “offline-to-online” trust transfer and those who did not, as well as between the family members of rural householders who experienced “offline-to-online” trust transfer and both rural householders and family members who did not undergo such trust transfer.

**Table 2:** Analysis of sample characteristics.

Sample characteristics	Rural householders who experienced trust transfer (n=551)	Family members who experienced trust transfer (n=969)	Rural householders who did not experience trust transfer (n=409)	Family members who did not experience trust transfer (n=668)	Mean differences (one) 1-3^a^	Mean differences (two) 2-3^b^	Mean differences (three) 2-4^c^
Online medical consultation	0.902	0.644	0.183	0.313	0.719^d^	0.461^d^	0.331^d^
Sex	1.379	1.520	1.347	1.485	0.032	0.173^d^	0.035
Age	50.486	44.132	35.694	46.681	14.792^d^	8.438^d^	–2.549^d^
Education	8.382	8.801	9.663	9.187	–1.280^d^	–0.862^d^	–0.386^e^
Occupation	0.053	0.050	0.017	0.072	0.036^d^	0.032^d^	–0.022^f^
Per capita disposable income	15,730.890	15,624.590	14,609.490	14,902.830	1121.403^e^	1015.108^e^	721.762^e^
Health status	2.299	2.280	2.039	2.168	0.260^d^	0.241^d^	0.112^d^
Home network access	0.653	0.671	0.589	0.593	0.064^e^	0.082^d^	0.078^d^
Number of home mobile phones and computers	3.143	3.149	2.606	2.666	0.524^d^	0.542^d^	0.482^d^
Whether health information is obtained from the internet	0.806	0.635	0.284	0.472	0.522^d^	0.351^d^	0.163^d^
Whether they recognize any acquaintances engaged in internet medicine	0.015	0.013	0.017	0.012	–0.003	–0.004	0.001
Distance to the nearest medical facility	13.504	13.223	14.195	14.126	–0.691	–0.972^e^	–0.903^e^
Satisfaction with medical technology during a final offline visit with a physician	4.060	3.320	2.905	3.025	1.155^d^	0.415^d^	0.294^d^
Satisfaction with the service attitude of the physician during the last offline visit	4.183	3.717	2.814	3.799	1.369^d^	0.903^d^	–0.082^e^
Satisfaction with frequently visited medical institutions	3.789	3.723	3.110	3.690	0.679^d^	0.613^d^	0.033

^a^The mean differences represent the disparity between the mean values of characteristics among householders who experienced “offline-to-online” trust transfer and those who did not.

^b^The mean differences depict the difference between the mean values of characteristics among family members whose householders experienced “offline-to-online” trust transfer and householders who did not experience such trust transfer.

^c^The mean differences denote the difference between the mean values of characteristics among family members whose householders experienced “offline-to-online” trust transfer and family members whose householders did not experience such trust transfer.

^d^Results of the “mean difference *t* test (2-tailed and paired)” are statistically significant at the 1% level.

^e^Results of the “mean difference *t* test” are statistically significant at the 5% level.

^f^Results of the “mean difference *t* test” are statistically significant at the 10% level.

### Propensity Score Estimation

The logit equation was used to estimate the propensity score of rural residents who experienced “offline-to-online” trust transfer (Table 3). The propensity of rural residents who experienced “offline-to-online” trust transfer was significantly related to their sociodemographic characteristics (*P*=.04), home network access (*P*=.002), and health care status (*P*<.001). Compared with other populations, older women with low-level education and better health status exhibited a higher likelihood of experiencing “offline-to-online” trust transfer. This inclination is particularly pronounced in populations whose occupations are internet related, those who primarily obtain health information from the internet, households with access to multiple computers and mobile phones, and individuals with high satisfaction levels toward offline medical institutions, including their medical technology and service attitude.

**Table 3:** Estimation of propensity score of the logistic model.

Variable^a^	Coefficient	Standard error	*z* score
Sex	0.346^b^	0.103	3.350
Age	0.012^c^	0.005	2.680
Education	–0.110^b^	0.018	–6.12
Occupation	0.940^b^	0.256	3.67
Per capita disposable income	0.00014^d^	7.870	1.900
Health status	0.576^b^	0.074	7.840
Home network access	0.221^c^	0.106	2.080
Number of home mobile phones and computers	0.439^b^	0.055	7.970
Whether health information is obtained from the internet	1.390^b^	0.105	13.25
Whether they recognize any acquaintances engaged in internet medicine	0.272	0.473	0.580
Distance to the nearest medical facility	–0.014^c^	0.007	–1.970
Satisfaction with medical technology during a final offline visit with a physician	0.460^b^	0.061	7.510
Satisfaction with the service attitude of the physician during the last offline visit	0.996^b^	0.068	14.730
Satisfaction with frequently visited medical institutions	0.281^b^	0.066	4.270
Constant term	–8.945^b^	0.573	–15.61

^a^The likelihood ratio statistic, pseudo *R*^2^, and observed value were 953.97, 0.287, and 2597, respectively.

^b^Significant at the statistical level of 1%.

^c^Significant at the statistical level of 5%.

^d^Significant at the statistical level of 10%.

### Balance Test

It is imperative to ensure that the matching results adhere to the balance hypothesis to validate the reliability of the propensity score. This entails controlling the standardized deviation of each covariate after model matching within 10%. The results of the balance test are presented in Table 4, where columns 3 and 4 show the mean values of the treated sample (rural residents who experienced “offline-to-online” trust transfer) and the control sample (rural residents who did not experience such trust transfer) before and after matching. Column 5 displays the standardized deviation, and columns 6 and 7 provide the *t* test results.

The results indicate that sex (*P*=.04), education (*P*<.001), occupation (*P*=.003), per capita disposable income (*P*=.03), and whether health information is obtained from the internet (*P*<.001) were significantly different between the prematched treated sample and the control sample. However, after matching, the differences were not significant (*P*=.95, .27, .10, .58, and .34, respectively), suggesting that these characteristics achieved a balance between the treated sample and the control sample. Moreover, with the exception of age, health status, the number of computers and mobile phones in the family, and individuals’ satisfaction with physicians’ technical and service attitude, the standardized deviation of other covariates in the treated sample and the control sample, after matching, was reduced and fell within the 10% range. This suggests that the disparities between the treated sample and the control sample were essentially eliminated, confirming the fulfillment of the equilibrium assumption.

**Table 4:** Balance test results of the treated and control samples.

Variable		Mean value		Standardized deviation	*t* test results	
		Treated sample	Control sample		*t* (*df*) value	*P* value
**Sex**						
	Before matching	1.474	1.415	12.0	2.890 (2596)	.004^a^
	After matching	1.473	1.485	–0.2	–0.070 (2596)	.95
**Age**						
	Before matching	45.916	42.637	27.7	6.710 (2596)	<.001
	After matching	45.910	47.247	–11.3	–3.110 (2596)	.002
**Education**						
	Before matching	8.704	9.423	–23.5	–5.910 (2596)	<.001^b^
	After matching	8.704	8.589	3.7	1.100 (2596)	.27
**Occupation**						
	Before matching	0.060	0.033	12.8	2.960 (2596)	.003^b^
	After matching	0.059	0.058	0.0	0.000 (2596)	>.99
**Per capita disposable income**						
	Before matching	15508	14897	9.2	2.190 (2596)	.03^a^
	After matching	15504	15381	1.8	0.550 (2596)	.58
**Health status**						
	Before matching	2.291	2.072	30.6	7.440 (2596)	<.001
	After matching	2.289	2.202	12.2	3.590 (2596)	<.001
**Home network access**						
	Before matching	0.653	0.597	11.7	2.830 (2596)	.005
	After matching	0.652	0.616	7.5	2.200 (2596)	.03
**Number of home mobile phones and computers**						
	Before matching	3.077	2.657	44.5	10.510 (2596)	<.001
	After matching	3.071	2.973	10.4	2.990 (2596)	.003
**Whether health information is obtained from the internet**						
	Before matching	0.689	0.347	72.8	17.630 (2596)	<.001^b^
	After matching	0.688	0.673	3.2	0.950 (2596)	.34
**Whether they recognize any acquaintances engaged in internet medical treatment**						
	Before matching	0.015	0.011	3.3	0.770 (2596)	.44
	After matching	0.015	0.008	6.1	1.910 (2596)	.06
**Distance to the nearest medical facility**						
	Before matching	13.378	14.235	–12.1	–2.950 (2596)	.003
	After matching	13.396	13.943	–7.7	–2.28 (2596)	.02
**Satisfaction with medical technology during a final offline visit with a physician**						
	Before matching	3.507	3.000	57.7	13.810 (2596)	<.001
	After matching	3.504	3.411	10.5	3.21 (2596)	.001
**Satisfaction with the service attitude of the physician during the last offline visit**						
	Before matching	3.931	3.234	83.7	21.030 (2596)	<.001
	After matching	3.928	4.060	–15.8	–4.920 (2596)	<.001
**Satisfaction with frequently visited medical institutions**						
	Before matching	3.738	3.425	38.9	9.600 (2596)	<.001
	After matching	3.738	3.802	–7.9	–2.52 (2596)	.01

^a^Significant at the statistical level of 5%.

^b^Significant at the statistical level of 1%.

### The Direct Effects of “Offline-to-Online” Trust Transfer on the Utilization of Online Medical Consultation of Rural Residents

The ATT regarding the impact of rural residents’ “offline-to-online” trust transfer on their online medical consultation was estimated using 4 propensity score matching methods (Table 5). The regression results revealed consistent findings across the 4 matching methods, with the ATT value being statistically significant at the 1% level. For rural residents who did not experience an “offline-to-online” trust transfer, their probability of utilizing online medical consultation ranged from 31% to 34%. By contrast, this probability increased to 70% for rural residents who experienced an “offline-to-online” trust transfer. The probability of utilizing online medical consultation among rural residents who experienced “offline-to-online” trust transfer was 37%-40% higher than that of residents who did not undergo such trust transfer. This finding suggests that “offline-to-online” trust transfer has a significant direct effect in promoting the utilization of online medical consultation for rural residents (*P*=.003).

The reliability of the results of processing effect estimation using the propensity score matching method depends on whether respondents accept that the processing factors are determined by the observable variables (ie, whether the conditional mean is independent). However, if the processing factors are determined by unobservable variables, hidden bias may occur, and the robustness of the estimation results could be compromised. To ensure that the estimation model does not overlook other crucial variables related to “offline-to-online” trust transfer and the utilization of online medical consultation among rural residents, we used the Markov matching method to test the robustness of the results (Table 6). By calculating heteroscedasticity and robust SE, we verified the robustness of the matching results.

**Table 5:** Impact of “offline-to-online” trust transfer of rural residents on how they utilize online medical consultation.

Matching method	Treated sample	Control sample	ATT^a,b^
K-nearest neighbor matching (k=1)	0.705	0.309	0.396^c^ (0.042)
K-nearest neighbor matching (k=4)	0.705	0.338	0.368^c^ (0.037)
Radius matching	0.705	0.329	0.377^c^ (0.033)
Nuclear matching	0.705	0.325	0.380^c^ (0.027)

^a^The SE obtained by the self-help method (repeated 200 times) is shown in brackets.

^b^ATT: average treatment effects on the treated.

^c^Significant at the statistical level of 1%.

**Table 6:** Robustness test for the “utilization of online medical consultation” variable.

Sample	Treated	Controls	Difference	SE	*t* statistics
Unmatched	0.706	0.218	0.488	0.018	26.680 (2596)
ATT^a^	0.706	0.371	0.335	0.030	11.290 (1404)
ATU^b^	0.218	0.556	0.338	0.025	13.630 (1191)
ATE^c^	N/A^d^	N/A	0.336	0.024	13.730 (2596)

^a^ATT: average treatment effects on the treated.

^b^ATU: average treatment effect on the uncontrol.

^c^ATE: average treatment effect.

^d^N/A: not applicable.

### The Family Spillover Effect of “Offline-to-Online” Trust Transfer on the Utilization of Online Medical Consultation of Rural Residents

We conducted a match between the family members of householders who experienced “offline-to-online” trust transfer and those of householders who did not experience such trust transfer. Additionally, matching was performed between the family members of householders who experienced “offline-to-online” trust transfer and those of householders who did not experience such trust transfer. This was done to further investigate the influence of rural families on individuals’ perceptions and behavior. The results indicated that the probability of family members of rural householders who experienced “offline-to-online” trust transfer utilizing online medical consultation was 28%-30% higher than that of rural householders who did not experience such trust transfer.

Furthermore, in comparison to family members of householders who did not experience “offline-to-online” trust transfer, the presence of “offline-to-online” trust transfer significantly promoted the utilization of online medical consultation by family members of householders who experienced such trust transfer (Tables 7 and 8). The results showed that the probability of family members of householders who experienced “offline-to-online” trust transfer utilizing online medical consultation was 25%-28% higher than that of family members of rural householders who did not experience such trust transfer. These findings suggest that rural householders have a family spillover effect on the utilization of online medical consultation by their family members.

**Table 7:** The family spillover effects of “offline-to-online” trust transfer: when comparing householders who did not undergo the “offline-to-online” trust transfer with those who did, the average treatment effects of such trust transfer on the utilization of online medical consultation by family members who experienced the trust transfer are examined.

Matching method	Treated sample	Control sample	ATT^a^	
K-nearest neighbor matching (k=1)	0.655	0.381	0.274^b^ (0.070)	
K-nearest neighbor matching (k=4)	0.655	0.365	0.290^b^ (0.071)	
Radius matching	0.655	0.368	0.287^b^ (0.067)	
Nuclear matching	0.655	0.368	0.287^b^ (0.064)	

^a^The SE obtained by the self-help method (repeated 200 times) is shown in brackets.

^b^Significant at the statistical level of 1%.

**Table 8:** The family spillover effects of “offline-to-online” trust transfer: the average treatment effects of “offline-to-online” trust transfer on the utilization of online medical consultation among family members who did not experience such trust transfer are compared with those who did not undergo “offline-to-online” trust transfer.

Matching method	Treated sample	Control sample	ATT^a^	
K-nearest neighbor matching (k=1)	0.609	0.334	0.275^b^ (0.047)	
K-nearest neighbor matching (k=4)	0.609	0.360	0.249^b^ (0.041)	
Radius matching	0.609	0.355	0.254^b^ (0.038)	
Nuclear matching	0.609	0.357	0.252^b^ (0.033)	

^a^The SE obtained by the self-help method (repeated 200 times) is shown in brackets.

^b^Significant at the statistical level of 1%.

### The Distributional Effect of “Offline-to-Online” Trust Transfer on the Utilization of Online Medical Consultation of Rural Residents

The results indicated that, among the 3 levels of education samples, the direct effect of “offline-to-online” trust transfer on the utilization of online medical consultation by rural residents was highest in the primary education sample. The family spillover effect of “offline-to-online” trust transfer on the utilization of online medical consultation by rural residents was highest in the secondary education sample. The probability of family members of householders who experienced “offline-to-online” trust transfer utilizing online medical consultation was 19%-34% higher than that of householders who did not experience such trust transfer (Tables 9 and 10).

**Table 9:** The distributional effects of “offline-to-online” trust transfer of rural residents with different education levels: the direct effect of “offline-to-online” trust transfer on the utilization of online medical consultation of rural residents with different education levels.^a^

Education level	ATT (K-nearest neighbor matching n=1)	ATT (K-nearest neighbor matching n=4)	ATT (radius matching)	ATT (nuclear matching)
Primary education	0.412^b^ (0.053)	0.398^b^ (0.049)	0.393^b^ (0.048)	0.396^b^ (0.043)
Secondary education	0.268^b^ (0.054)	0.337^b^ (0.050)	0.326^b^ (0.045)	0.342^c^ (0.040)
Higher education	0.444 (0.228)	0.417 (0.193)	0.465 (0.358)	0.419 (0.221)

^a^The SE obtained by the automatic method (repeated 200 times) is shown in brackets.

^b^Significant at the statistical level of 5%.

^c^Significant at the statistical level of 1%.

**Table 10:** The distributional effects of “offline-to-online” trust transfer of rural residents with different education levels: the spillover effect of “offline-to-online” trust transfer on the utilization of online medical consultation of rural residents with different education levels.^a^

Education level	ATT (K-nearest neighbor matching n=1)	ATT (K-nearest neighbor matching n=4)	ATT (radius matching)	ATT (nuclear matching)
Primary education	0.192 (0.126)	0.246^b^ (0.117)	0.181 (0.120)	0.232^b^ (0.106)
Secondary education	0.189^b^ (0.096)	0.343^c^ (0.097)	0.209^b^ (0.090)	0.288^c^ (0.085)

^a^The SE obtained by the automatic method (repeated 200 times) is shown in brackets.

^b^Significant at the statistical level of 1%.

^c^Significant at the statistical level of 5%.

## Discussion

### Principal Findings

Trust can indeed be transferred between related entities [61], not only within the same channel but also between different channels [62]. We confirmed that “offline-to-online” trust transfer was an effective mechanism to assist rural residents in building trust in online medical consultation. Additionally, it is important to note that the occurrence of trust transfer necessitates a third party endowed with mutual trust between the “source” and “target” of trust transfer [27]. Functioning as an effective intermediary, this third party facilitates “source-target” trust transfer at the interpersonal level. In our context, offline physicians who are familiar with individuals can be considered as the “source” of trust transfer, the “target” of trust transfer being the online medical consultation provided by these offline physicians. The online medical consultation delivered or recommended by offline physicians on the online platform enhances the perceived similarity between channels and, consequently, the individual’s trust in the online medical consultation.

Moreover, in social and economic interactions marked by high uncertainty and dependence, the existence of robust trust becomes a pivotal factor in augmenting interaction [21]. This trust plays a vital role in helping individuals overcome perceptions of risk and insecurity, thereby influencing their behavioral intentions [63]. Our findings indicate that the trust built through “offline-to-online” trust transfer was sufficiently robust to enhance rural residents’ intention to utilize online medical consultation. This trust proved instrumental in overcoming significant uncertainties and risk expectations related to service quality, personal privacy, and property safety inherent in this non–face-to-face service mode. Additionally, our research revealed that household heads exert a nonnegligible influence on the opinion formation and behavioral decision-making of other family members [54]. When household heads undergo an “offline-to-online” trust transfer and actively engage with online medical consultation, family members are likely to be influenced by the household head, leading to the formation of a positive perception and willingness to utilize online medical consultation. This implies that the impact of “offline-to-online” trust transfer on the utilization of online consultation services among Chinese rural residents could be magnified through the family spillover effect. Moreover, by offering family assistance [64], we can facilitate the development of trust in online medical consultation and enhance its utilization among rural residents.

Additionally, we observed that the impact of “offline-to-online” trust transfer on the utilization of online medical consultation among rural residents is more pronounced with lower levels of education. This finding contrasts with the conclusions of existing studies, which suggest that individuals with higher levels of education are more likely to embrace emerging concepts [32]. This may be attributed to the tendency of Chinese individuals to be more risk-averse compared with people in other countries [65]. They may be inclined to pay closer attention to various risk factors, such as security and privacy, when choosing online medical consultation services. Furthermore, individuals with higher levels of education often possess higher cognitive abilities and insights [17], which may increase their awareness of the mentioned risks, subsequently leading to heightened levels of anxiety, depression, and other negative emotions [66]. These perceptions of risk and negative emotions may contribute to an increased sense of risk aversion and, in conjunction, counteract the positive effects of trust, thereby hindering the individual’s utilization of online medical consultation.

Furthermore, our findings indicate that rural residents who are female, older, in better health, and possess multiple computers and mobile phones are more likely to experience “offline-to-online” trust transfer. Additionally, increased satisfaction with offline physicians’ technical skills, service attitude, and health care institutions correlates with a higher likelihood of experiencing “offline-to-online” trust transfer. This pattern could be attributed to the fact that older women tend to exhibit higher levels of social trust, making them more predisposed to trust-emerging health care service modes [67] and experience “offline-to-online” trust transfer. Similarly, individuals in relatively good health may be more inclined to try online medical consultation, especially for the diagnosis and treatment of minor and common diseases, consequently experiencing “offline-to-online” trust transfer when engaging with such services. Home internet devices contribute to creating a supportive environment that facilitates individuals’ access to online medical services [68]. Furthermore, a positive experience with technology and services provided by offline health care institutions and physicians can significantly enhance an individual’s trust in offline physicians [8], thereby fostering their experience of “offline-to-online” trust transfer.

### Limitations

Our study has some limitations. First, we used a cross-sectional survey to investigate rural residents’ “offline-to-online” trust transfer and their utilization of online medical consultation. The use of contemporaneous data only allows us to discern the correlation between these variables, not establishing a causal relationship. Second, the propensity score matching method is more adept at controlling the heterogeneity of observable factors. However, individuals’ utilization of online medical consultation may be influenced by unobservable variables such as personal risk preference, potentially introducing “hidden bias” into the estimation.

### Conclusions

The “offline-to-online” trust transfer emerges as a dependable method for fostering rural residents’ trust in online medical consultation. Enhancing patient satisfaction with offline health care services could play a pivotal role in promoting “offline-to-online” trust transfer. Improving rural residents’ experience of “offline-to-online” trust transfer proves to be an effective strategy to encourage their utilization of online medical consultation services, particularly for populations with lower levels of education. Additionally, the influence of family dynamics in this process cannot be overlooked. Hence, the example of online medical consultation, being a critical component of mHealth and uHealth services, underscores the importance of such services in future promotions. First, offline physicians should take on the role of trust transmission to facilitate rural residents’ “offline-to-online” trust transfer. Advocating for a “patient-oriented” physician-patient service mode, strengthening communication between physicians and patients, fostering a harmonious physician-patient relationship, and bolstering trust in offline physicians among rural residents are crucial. Encouraging offline physicians to actively introduce and recommend online medical platforms can help compensate for rural residents’ lack of experience and limited access to information channels. Second, families should assume a significant role in facilitating the family spillover effect and amplifying the impact of “offline-to-online” trust transfer on rural residents’ utilization of mHealth and uHealth services. Leveraging familial bonds and geographic proximity, efforts can be made to enhance awareness and understanding of service content, utilization benefits, and platform systems among rural residents. Building trust in mHealth and uHealth services among family householders and even village leaders is crucial. Encouraging them to actively recommend mHealth and uHealth services to their family members and the broader community can significantly contribute to promoting these services. Additionally, it is important to note that the effectiveness of “offline-to-online” trust transfer may not be as pronounced for individuals with higher levels of education when it comes to building trust in mHealth and uHealth services. In future research, it is imperative to explore and identify effective strategies for promoting trust and utilization of mHealth and uHealth services, with a specific focus on individuals with higher levels of education.

## Appendix

Multimedia Appendix 1 The trust transfer game and the definition of the “offline-to-online” trust transfer.

## Abbreviations

ATE: average treatment effect
ATT: average treatment effects on the treated
ATU: average treatment effect on the uncontrol
mHealth: mobile health
uHealth: ubiquitous health

## References

[1] Li X, Lu J, Hu S, Cheng K, De Maeseneer J, Meng Q, Mossialos E, Xu DR, Yip W, Zhang H, Krumholz HM, Jiang L, Hu S (2017). The primary health-care system in China. The Lancet.

[2] Zhong H (2011). Effect of patient reimbursement method on health-care utilization: evidence from China. Health Econ.

[3] Huang M, Zhang H, Gu Y, Wei J, Gu S, Zhen X, Hu X, Sun X, Dong H (2019). Outpatient health-seeking behavior of residents in Zhejiang and Qinghai Province, China. BMC Public Health.

[4] Zhang S, Sun K, Zheng R, Zeng H, Wang S, Chen R, Wei W, He J (2021). Cancer incidence and mortality in China, 2015. Journal of the National Cancer Center.

[5] Shadmi E, Chen Y, Dourado I, Faran-Perach I, Furler J, Hangoma P, Hanvoravongchai P, Obando C, Petrosyan V, Rao KD, Ruano AL, Shi L, de Souza LE, Spitzer-Shohat S, Sturgiss E, Suphanchaimat R, Uribe MV, Willems S (2020). Health equity and COVID-19: global perspectives. Int J Equity Health.

[6] Li X, Krumholz HM, Yip W, Cheng KK, De Maeseneer J, Meng Q, Mossialos E, Li C, Lu J, Su M, Zhang Q, Xu DR, Li L, Normand ST, Peto R, Li J, Wang Z, Yan H, Gao R, Chunharas S, Gao X, Guerra R, Ji H, Ke Y, Pan Z, Wu X, Xiao S, Xie X, Zhang Y, Zhu J, Zhu S, Hu S (2020). Quality of primary health care in China: challenges and recommendations. The Lancet.

[7] Chen Y, Yin Z, Xie Q (2014). Suggestions to ameliorate the inequity in urban/rural allocation of healthcare resources in China. Int J Equity Health.

[8] Wu Y, Zhang Q, Huang Y, Qiu S (2022). Seeking medical services among rural empty-nest elderly in China: a qualitative study. BMC Geriatr.

[9] Yang M, Jiang J, Kiang M, Yuan F (2022). Re-examining the impact of multidimensional trust on patients' online medical consultation service continuance decision. Inf Syst Front.

[10] Jiang J, Yang M, Kiang M, Cameron A (2021). Exploring the freemium business model for online medical consultation services in China. Information Processing & Management.

[11] Lai Y, Chen S, Li M, Ung COL, Hu H (2021). Policy interventions, development trends, and service innovations of internet hospitals in China: documentary analysis and qualitative interview study. J Med Internet Res.

[12] Banbury A, Roots A, Nancarrow S (2014). Rapid review of applications of e-health and remote monitoring for rural residents. Aust J Rural Health.

[13] Bekeshova E (2020). Problems in the organization of primary health care for the rural population at the current stage. BSP.

[14] Zhang T, Lu B, Wang X (2022). Urban-rural disparity in cognitive performance among older Chinese adults: explaining the changes from 2008 to 2018. Front Public Health.

[15] Tennant B, Stellefson M, Dodd V, Chaney B, Chaney D, Paige S, Alber J (2015). eHealth literacy and Web 2.0 health information seeking behaviors among baby boomers and older adults. J Med Internet Res.

[16] Qiu Y, Ren W, Liu Y, Yin P, Ren J (2019). Online health information in a rural residential population in Zhejiang Province, China: a cross-sectional study. BMJ Open.

[17] Evangelista L, Steinhubl SR, Topol EJ (2019). Digital health care for older adults. The Lancet.

[18] Ma C, Song Z, Zong Q (2021). Urban-rural inequality of opportunity in health care: evidence from China. Int J Environ Res Public Health.

[19] Zhou X, Chen L (2021). Digital health care in China and access for older people. The Lancet Public Health.

[20] Martins Van Jaarsveld G (2020). The effects of COVID-19 among the elderly population: a case for closing the digital divide. Front Psychiatry.

[21] Li Y(, James L, McKibben J (2016). Trust between physicians and patients in the e-health era. Technology in Society.

[22] Stewart KJ (2003). Trust transfer on the world wide web. Organization Science.

[23] Zhang KZ, Zhao SJ, Cheung CM, Lee MK (2014). Examining the influence of online reviews on consumers' decision-making: a heuristic–systematic model. Decision Support Systems.

[24] van Velsen L, Flierman I, Tabak M (2021). The formation of patient trust and its transference to online health services: the case of a Dutch online patient portal for rehabilitation care. BMC Med Inform Decis Mak.

[25] Cao Y, Zhang J, Ma L, Qin X, Li J (2020). Examining user's initial trust building in mobile online health community adopting. Int J Environ Res Public Health.

[26] Meng F, Guo X, Peng Z, Lai K, Zhao X (2019). Investigating the adoption of mobile health services by elderly users: trust transfer model and survey study. JMIR Mhealth Uhealth.

[27] Zhang KZ, Gong X, Chen C, Zhao SJ, Lee MK (2019). Spillover effects from web to mobile payment services. INTR.

[28] Leech AA, Lin P, D'Cruz Brittany, Parsons SK, Lavelle TA (2023). Family spillover effects: are economic evaluations misrepresenting the value of healthcare interventions to society?. Appl Health Econ Health Policy.

[29] Basu A, Meltzer D (2005). Implications of spillover effects within the family for medical cost-effectiveness analysis. J Health Econ.

[30] Sato A (2012). Does socio-economic status explain use of modern and traditional health care services?. Soc Sci Med.

[31] Zaefarian G, Kadile V, Henneberg SC, Leischnig A (2017). Endogeneity bias in marketing research: problem, causes and remedies. Industrial Marketing Management.

[32] Pei Z, Pischke J, Schwandt H (2018). Poorly measured confounders are more useful on the left than on the right. Journal of Business & Economic Statistics.

[33] Caliendo M, Kopeinig S (2008). Some practical guidance for the implementation of propensity score matching. Journal of Economic Surveys.

[34] Wang P, Liu Q, Qi Y (2014). Factors influencing sustainable consumption behaviors: a survey of the rural residents in China. Journal of Cleaner Production.

[35] Klaes M, Sent EM (2005). A conceptual history of the emergence of bounded rationality. History of Political Economy.

[36] Bhattacharya R, Devinney TM, Pillutla MM (1998). A formal model of trust based on outcomes. AMR.

[37] Xu Y, Yang Z, Jiang H, Sun P (2022). Research on patients' willingness to conduct online health consultation from the perspective of web trust model. Front Public Health.

[38] Hogarth RM, Betsch T, Haberstroh S (2005). Deciding analytically or trusting your intuition? The advantages and disadvantages of analytic and intuitive thought. The Routines of Decision Making.

[39] Kruglanski AW, Gigerenzer G (2011). Intuitive and deliberate judgments are based on common principles. Psychol Rev.

[40] Sharma S, Menard P, Mutchler LA (2017). Who to trust? Applying trust to social commerce. Journal of Computer Information Systems.

[41] Shan C, Lu Y (2009). How offline-to-online trust transference affect the foundation of online banking initial trust: an empirical investigation.

[42] Delgado-Márquez BL, Hurtado-Torres NE, Aragón-Correa JA (2012). The dynamic nature of trust transfer: mMeasurement and the influence of reciprocity. Decision Support Systems.

[43] Kovacs Roxanne J, Lagarde Mylene, Cairns John (2019). Measuring patient trust: comparing measures from a survey and an economic experiment. Health Econ.

[44] Berg J, Dickhaut J, McCabe K (1995). Trust, reciprocity, and social history. Games and Economic Behavior.

[45] Sheppard BH, Jon H, Warshaw PR (1988). The theory of reasoned action: a meta-analysis of past research with recommendations for modifications and future research. Journal of Consumer Research.

[46] Ma Z, Wang L, Li Eph, Zhang J (2021). Inter‐ versus intra‐channel trust transfer on an online‐to‐offline (O2O) platform. Can J Adm Sci.

[47] Venkatesh, Morris, Davis, Davis (2003). User acceptance of information technology: toward a unified view. MIS Quarterly.

[48] Lu J, Yao JE, Yu C (2005). Personal innovativeness, social influences and adoption of wireless internet services via mobile technology. The Journal of Strategic Information Systems.

[49] Venkatesh V, Davis FD (2000). A theoretical extension of the technology acceptance model: four longitudinal field studies. Management Science.

[50] Harm B, Marianne A (2000). The cycle of socialization. Readings for Diversity and Social Justice.

[51] Rothbaum F, Rosen K, Ujiie T, Uchida N (2002). Family systems theory, attachment theory, and culture. Fam Process.

[52] Deal KH (2007). Psychodynamic theory. ASW.

[53] Turner JH (2012). Contemporary Sociological Theory.

[54] Lien NH, Westberg K, Stavros C, Robinson LJ (2018). Family decision-making in an emerging market: tensions with tradition. Journal of Business Research.

[55] Peek ST, Wouters EJ, van Hoof J, Luijkx KG, Boeije HR, Vrijhoef HJ (2014). Factors influencing acceptance of technology for aging in place: a systematic review. Int J Med Inform.

[56] Evans JSBT (2011). Heuristic and analytic processes in reasoning. Br J Psychol.

[57] Nan (2011). Capturing bottom-up information technology use processes: a complex adaptive systems model. MIS Quarterly.

[58] Yin MZ, Yu WP, Zhou TM (2017). An empirical study on the influencing factors of university library users' continuous willingness to use the We Chat. Library Theory and Practice.

[59] Shen ZW, Jiang YS (2008). Determinants of health and family medical expenditure among rural residents in Western China: based on a survey of farmers in Sichuan and Shaanxi. Agricultural Technology and Economy.

[60] Rosenthal CJ, Marshall VW (1986). The head of the family: social meaning and structural variability. Canadian Journal of Sociology/Cahiers canadiens de sociologie.

[61] Belanche D, Casaló LV, Flavián C, Schepers J (2014). Trust transfer in the continued usage of public e-services. Information & Management.

[62] Lee KC, Kang I, McKnight DH (2007). Transfer from offline trust to key online perceptions: an empirical study. IEEE Trans Eng Manage.

[63] Harrison McKnight D, Choudhury V, Kacmar C (2002). The impact of initial consumer trust on intentions to transact with a web site: a trust building model. The Journal of Strategic Information Systems.

[64] Bianchi C (2021). Exploring how internet services can enhance elderly well-being. JSM.

[65] Hsee CK, Weber EU (1999). Cross-national differences in risk preference and lay predictions. J Behav Decis Making.

[66] Butler G, Mathews A (1987). Anticipatory anxiety and risk perception. Cogn Ther Res.

[67] Chen-Xi HE, Zhao X (2015). Research on the satisfaction degree and its influencing factors of agricultural extension services: based on the survey data of 1033 farmersJ. Research of Agricultural Modernization.

[68] Morgan J, Ravindran S (2014). An examination of home internet and mobile device use in the U.S. IJIKM.

