# Supplementary material for: The Effect of “Offline-to-Online” Trust Transfer on the Utilization of Online Medical Consultation Among Chinese Rural Residents: Experimental Study
Source: J Med Internet Res. 2023 Dec 26;25:e43430. doi: 10.2196/43430 (PMC10777280; doi:10.2196/43430)
Supplement: Multimedia Appendix 1 [file jmir_v25i1e43430_app1.docx]

**Multimedia Appendix 1**

**The trust transfer game that is used to investigate the status of individuals’ “offline-to-online” trust transfer**

The trust transfer game is a behavioral economics experiment from the trust game. They all contains a series of simple decision-making tasks that are carried out by two types of people (or organizations): the truster and the trustee, in order to measures the trust level of the truster (the issuer of the trust) to the trustee (the recipient of the trust) [1]. During the experiment, the truster accepts a monetary donation and chooses how much of the donation, such as keeping it all, donating half, or donating all to trustee. To measures the trust level of the truster (the issuer of the trust) to the trustee (the recipient of the trust). The amount remit by the truster is interpret as a measure of their trust in the trustee [2]. This because that only when the truster expects the trustee to return a portion of their donation, rather than using the money for their own use, will they give the money [3].

Different from the trust game, which involved two individuals (or two organizations), that was one truster and one trustee, the trust transfer game extend the experimental subject to four individuals (or four organizations), including one truster and three types of trustee. The three trustees were the person trusted by the truster (the trustee), the person who was associated with a person trusted by the truster (the third trustee), and the person who was completely unknown to the truster (the stranger) [4]. Also, the trust transfer game mainly used to measure the trust level of the truster to the thrid trustee because he/she trust the trustee.

In this study, each participant was required to play three simple decision-making tasks with three types of healthcare service providers: the offline doctor whom this individual is familiar (the trustee), the online medical consultation where this physician worked or that the physician recommended (the third trustee), and the online medical platform where this physician did not work and did not recommend (the stranger). These three different types represent the psychological distance between this individual and three different providers, namely, an offline physician with whom this individual was familiar (the trustee) < an online medical platform where this physician worked or that the physician recommended (the third trustee) < an online medical platform where this physician did not work and did not recommend(the stranger).

The experimental procedure are as follows:

First, we asked participants whether they know online medical consultation before the survey. For those who do not know online medical consultation, we introduced it by image-text in the survey, and demonstrated the process of seeking diagnosis and treatment services on the online medical consultation. After making sure respondents understand the meaning of online medical consultation were, we conducted the trust transfer game and asked individuals if they would like to use online medical consultation.

Second, during the game, ① we give each participant the same initial consumption amount (RMB 300), and told them the money was used for transactions with three types of healthcare service providers. ②We will inform the participant that 50% of the amount returned will be rewarded as game reward. ③for each investigator, we will distribute three envelopes labeled with three types of trustees (an offline physician with whom this individual is familiar, an online medical platform where this physician worked or that the physician recommended, and an online medical platform where this physician did not work and did not recommend), and each investigator can put any amount (0~300RMB) into the corresponding envelope according to their own wishes.

Third, the management recorded the amount spent in each participant's envelope, and each participant received 50 yuan as a reward after the experiment was completed.

It should be recognized that the experimental reliability and validity are ensured from two aspects. First, prior to the game, the sentences in the game were repeatedly evaluated and checked to avoid "self-contradictory" and “beyond understanding” problems. We also trained the experimenters to ensured that they played the game objectively without hints or lead words. Second, during the game, each participant performs the amount decision process in a closed environment to reduce the influence of social norms and others on the amount sent. Meanwhile, the experimental treatment adopted by each participant (e.g., the experimental requirements, experimental rewards, etc.) are consistent, which could avoid interference from the external environment on the experimental results to the maximum extent.

**The definition of the “offline-to-online” trust transfer**

Offline-and-online trust transfer refers to the process of transferring trust from trusted offline entities to unknown online entities, highlighting the potential for establishing trust through personal cognition[5]. In the context of this study, "offline-to-online trust transfer" refers specifically to an individual build trust in online medical consultation services by transferring their trust from offline physicians to the online medical consultation services provided/recommended by these physicians.

Assuming that the truster is label as l and the three types of trustee are label as 2.

$x_{1 2}^{jd(j)}$ represents the amount of money that the jth truster paid to the jth trustee;

$y_{1 2}^{jd(j)}$ represents the amount of money that the jth truster paid to the jth third trustee;

$z_{1 2}^{jd(j)}$ represents the amount of money that the jth truster paid to the jth stranger.

Among them, $0\leq x_{1 2}^{jdip(j)}$,$y_{1 2}^{jdip(j)}$,$z_{1 2}^{jdip(j)}\leq3$00*;*$x_{1 2}^{jdip(j)}$*,*$y_{1 2}^{jdip(j)}$*,*$z_{1 2}^{jdip(j)}\in N$*.*We defined an individual experience the “offline-to-online” trust transfer when the truster is willing to pay money to the third trustee, and the amount is higher than the amount paid to the stranger, that is $0\leq z_{1 2}^{jdip(j)}\leq y_{1 2}^{jdip(j)}\leq x_{1 2}^{jdip(j)}\leq300$. The “offline-to-online” trust transfer is maximized when the amount received by the third trustee and the trustee is at least equal, this defined as an individual experience the compete “offline-to-online” trust transfer, that is $0\leq z_{1 2}^{jdip(j)}\leq y_{1 2}^{jdip(j)}$= $x_{1 2}^{jdip(j)}\leq300$.

**The truster**: The 2597 rural residents random sample by multi-stage stratified random sampling.

**The trustee**: The offline doctor whom this individual is familiar.

**The third trustee:** The online medical platform where this physician worked or that the physician recommended.

**The stranger:** The online medical platform where this physician did not work and did not recommend.

**The sampling procedures**

This study used a multi-stage stratified random sampling method to survey rural residents in China from July to September 2021. In the first stage, according to the regional economic development level released by the National Bureau of Statistics of the China in June 2011, the provinces (autonomous regions, municipalities directly under the Central Government) in the Chinese Mainland were divided into the eastern, central, western and northeastern regions (excluding Hong Kong, Macao and Taiwan), and a sample province (autonomous region, municipality directly under the Central Government) (Jiangsu Province, Shanxi Province, Gansu Province, Jilin Province) was selected from the four regions using the random number table method. In the second stage, according to the regional and urban-rural division code issued by the National Bureau of Statistics of the China in November 2020, two Prefecture-level city (regions, autonomous prefectures, etc.) were selected from four sample provinces using the random number table method, and a total of eight Prefecture-level city (regions, autonomous prefectures, etc.) were selected. In the third stage, two municipal districts (County-level city, counties, autonomous counties, etc.) were selected from eight Prefecture-level city (regions, autonomous prefectures, etc.) with the random number table method, a total of 16 municipal districts (County-level city, counties, autonomous counties, etc.). In the fourth stage, 32 townships and towns were selected from 16 municipal districts (County-level city, county, autonomous county, etc.) by random number table. In the fifth stage, the random number table method was also used to selected 2 administrative villages from 32 townships and towns, totaling 64 administrative villages. In the sixth stage, each rural household was numbered within the sample village, and 15 rural households were randomly selected from each sample village using the random number table method. The investigation was approved by the Institutional Ethical Review Board of Xi’an Jiaotong University and informed consent was obtained from all participants. A total of 960 rural households (2597 rural residents) were surveyed, and data collection was conducted on family members aged 18 and above in each household.”
